# Effect of acupuncture on Hashimoto thyroiditis: A systematic review and meta-analysis

**DOI:** 10.1097/MD.0000000000037326

**Published:** 2024-03-01

**Authors:** Xiaohui Wang, Yu Li, Hai Xie, Zhicheng Dai, Limin Ma, Xinying Zhu, Tongxia Zhan

**Affiliations:** a School of Nursing, Weifang Medical University, Weifang, China; b Affiliated Hospital of Weifang Medical University, Weifang, China; c Department of Clinical Medicine of Weifang Medical University, Weifang, China; d Institute of Plastic Surgery, Weifang Medical University, Weifang, China.

**Keywords:** acupuncture, hashimoto thyroiditis, levothyroxine sodium tablet

## Abstract

**Background:**

Hashimoto thyroiditis (HT) is a common autoimmune thyroid disease for which there is no specific treatment. Oral levothyroxine sodium tablets significantly improved thyroid function but did not promote a reduction in thyroid-related antibody concentrations. Acupuncture can improve clinical symptoms and thyroid function in HT patients, reduce serum TPOAb and TGAb levels in HT patients, and improve patients’ quality of life.

**Methods:**

We conducted a systematic review and meta-analysis to evaluate the effect of acupuncture versus levothyroxine sodium tablets on Hashimoto thyroiditis. We searched Web of Science, Embase, China National Knowledge Infrastructure, WanFang, VIP, SinoMed and the Cochrane Central Registry of Controlled Trials to identify candidate randomized controlled trials (RCTs).

**Results:**

A total of 1020 patients participated in 14 randomized controlled trials. The results of meta-analysis showed that acupuncture regulated TPOAb content (mean difference [MD] = −63.18, 95%CI = −91.73 to −34.62, *P* < .00001), TGAb content (MD = −68.56, 95%CI = −101.55 to −35.57, *P* < .00001), serum free triiodothyronine (FT3) content (MD = 0.74, 95%CI = 0.20 to 1.27, *P* < .00001), serum free thyroxine (FT4) content (MD = 1.10, 95%CI = 0.29 to 1.92, *P *< .00001), TSH content (MD = −2.16, 95%CI = −3.14 to −1.19, *P* < .00001) had a significant effect.

**Conclusion:**

Compared with levothyroxine sodium tablets alone, acupuncture can significantly regulate the contents of TPOAb, TGAb, FT3, FT4 and TSH.

## 1. Introduction

Hashimoto thyroiditis is a prevalent autoimmune disorder characterized by the production of autoantibodies that target thyroid-specific antigens.^[1]^ In the middle and late stages of the disease, thyroid tissue is destroyed and hypothyroidism often occurs,^[2]^ which seriously affects the physiological function and quality of life of patients. At present, the main clinical treatment for Hashimoto thyroiditis is oral levothyroxine sodium tablets, but some patients do not respond well to drug treatment, long-term drug use may also induce cardiovascular system, bone metabolism and other related complications.^[3]^ Acupuncture is a method of treatment through the use of needles, moxibustion and ear needles on the body. Previous studies have shown that acupuncture as a small but effective alternative therapy, has the effect of regulating thyroid hormone^[4]^and activating meridians, and this kind of treatment can relieve the side effects of taking drugs to a certain extent and is a beneficial choice for the treatment of thyroiditis in clinic.

According to the data of Ren Haitao et al,^[5]^ compared with oral selenium yeast tablets, the cervical soft tissue can be loosened by massage and acupuncture, which can improve the thyroid hormone levels, autoantibody levels, and the tissue structure of the diseased thyroid gland. According to the research of Xia Yong et al,^[6]^ moxibustion combined with oral thyroid hormone can improve clinical symptoms and thyroid function in Hashimoto thyroiditis patients, which is superior to oral thyroid function alone. Acupuncture may act on the hypothalamic-pituitary-thyroid axis through neuro-endocrine-immune system regulation. Acupuncture regulates the nervous system by stimulating the corresponding points on the body surface to generate somatosensory stimulation, which is converted into bioelectrical stimulation by the body.^[7]^ After the electrical stimulation activates the brainstem - reticular system in the body, it reaches the hypothalamus, stimulates the cerebral cortex and regulates the thyroid hormone level.^[8]^

To evaluate the effect of acupuncture on the efficacy of patients with Hashimoto thyroiditis, we included more outcome indicators, includes Thyroid Peroxidase Antibody (TPOAb), Thyroglobulin Antibody (TGAb), Serum Free Triiodothyronine (FT3), Serum Free Thyroxine (FT4), Thyroid Stimulating Hormone (TSH). Secondary outcome measures include serum anti-thyroid microsomal antibody (MCA), serum anti-thyroid peroxidase antibody (TGA), Hospital Anxiety Scale (HADS-A), Hospital Depression Scale (HADS-D) and Thyroid Iodine Uptake. Acupuncture can improve the clinical symptoms of Hashimoto thyroiditis patients, regulate thyroid function, and enhance the quality of life of patients.^[9]^ However, due to the limited number and quality of studies, there is still a lack of high-quality systematic evaluation evidence. Therefore, this study adopted meta-analysis method to collect randomized controlled trials on the treatment of Hashimoto thyroiditis in recent years, and thyroid drugs were used as the control group, so as to evaluate the efficacy and safety of acupuncture and moxibustion and provide evidence-based medical evidence for the treatment of thyroiditis by acupuncture.

## 2. Materials and methods

This systematic review and meta-analysis were designed in line with the criteria suggested by the Cochrane Collaboration. We used the preferred items for systematic review and meta-analysis (PRISMA) criteria to guide the reporting of the results.^[10]^ As this study was based on published data, the permissible consent from participants was not applicable. The meta-analysis was registered with the PROSPERO database (registration number CRD42023421600).

### 2.1. Literature search strategy

We conducted a systematic search of the literature to identify all randomized controlled trials involving the use of acupuncture to intervene in patients with Hashimoto thyroiditis. We searched the Web of Science (from 1946 to April 2023), PubMed (from 1966 to April 2023), Embase (from 1974 to April 2023), China National Knowledge Infrastructure (from 1976 to April 2023), Wanfang Data (from 1990 to April 2023), VIP (from 1992 to April 2023), SinoMed (from 1990 to April 2023) and the Central Cochrane Registry of Controlled Trials (from 1997 to April 2023) to identify language-free studies that might qualify. The following medical terms were used for the search: “Hashimoto’ s thyroiditis” or “chronic lymphocytic thyroiditis” or “autoimmune thyroiditis” or “Hashimoto’ s disease” and “Acupuncture” or “Acupuncture treatment” or “Moxibustion.”

### 2.2. Inclusion and exclusion criteria

The inclusion criteria of trials were as follows: The diagnosis criteria is a combination of the presence of serum antibodies against thyroid antigens and appearance on thyroid sonogram according to a review and the diagnosis criteria in Chinese clinical guidelines.^[11]^ If thyroid ultrasound indicates diffuse enlargement of the thyroid and reduced echogenicity of glands in a patient with elevated serum thyroid-associated antibody, the clinical diagnosis of HT can be established.^[12]^ Participants were diagnosed with Hashimoto thyroiditis, with or without hypothyroidism.

Individuals will be excluded from the study if they meet any of the following criteria: a history of thyroid surgery; a history of mental illness; conference proceedings and abstracts without full RCTs information, some studies with incomplete data; duplicates of published studies.

Included randomized controlled trials contained at least one of the following outcome measures: TPOAb, TGAb, FT3, FT4, TSH. Also included are MCA, TGA, HADS-A, HADS-D and Thyroid Iodine Uptake. In these included randomized controlled trials, acupuncture was used in the experimental group and levothyroxine sodium tablets were used in the control group.

### 2.3. Data extraction

In this study, 2 researchers screened through the literature independently and completed the extraction of data. The extracted results were then a cross-checked. Disagreements were resolved through discussion and judged by a third investigator. For literature that lacked original data, we tried to get in touch with the authors to obtain the raw data, otherwise, the studies were excluded. The information extracted included first author name, year of publication, country, sample size, intervention details, age, male-to-female ratio, course of disease, point selection, key elements of bias risk assessment, and outcome indicator data.

### 2.4. Quality and risk of bias assessment

The quality of all included studies was assessed by the JADAD scale, which assessed 4 aspects, including random sequence generation, random hiding, blinding, and exit. The tool is evaluated on a scale of 1 to 7, with ratings of 1 to 3 considered as low-quality literature and ratings of 4 to 7 considered as high-quality literature. We used the Cochrane Bias tool for RCTs to assess the risk of bias in the included literature, including 7 assessments: sequence generation, assignment hiding, subject blindness, outcome evaluators, exit and loss of follow-up, incomplete outcome data, and selective outcome reporting. The included literature was assessed as low risk, high risk, or unclear. Two independent reviewers conducted the assessment, and any discrepancies were resolved through negotiation with a third researcher.

### 2.5. Outcomes

Meta-analysis of 10 outcome indexes was conducted through literature integration. The primary outcome index includes Thyroid Peroxidase Antibody (TPOAb), Thyroglobulin Antibody (TGAb), Serum Free Triiodothyronine (FT3), Serum Free Thyroxine (FT4), Thyroid Stimulating Hormone (TSH). Secondary outcome measures include Serum Anti-thyroid Microsomal Antibody (MCA), Serum Anti-thyroid Peroxidase Antibody (TGA), Hospital Anxiety Scale (HADS-A), Hospital Depression Scale (HADS-D) and Thyroid Iodine Uptake.

### 2.6. Statistical analysis

Data was analyzed by Revman 5.3 software. Results for continuous data are expressed as mean difference (MD) and 95% confidence interval (CI). The χ^2^ and *I*^2^ tests were used to evaluate the heterogeneity of studies. A sensitivity analysis referred to the re-meta-analysis of data after sequentially removing single studies and compared the results after elimination with the original results. Subgroup analysis was performed to identify the effects of acupuncture method, JADAD score, duration of treatment, whether subjects had hypothyroidism, and date of publication were affected. For 14 articles, the data was expressed as a median. Therefore, the algorithm of Hozo et al^[13]^ was used to estimate the weighted mean and standard deviation. Similarly, the test for overall effects determined the result by the *P* value magnitude, when *P* < .05 the data was considered to be statistically significant. Publication bias was determined by funnel plots.

## 3. Result

**Figure 1.**
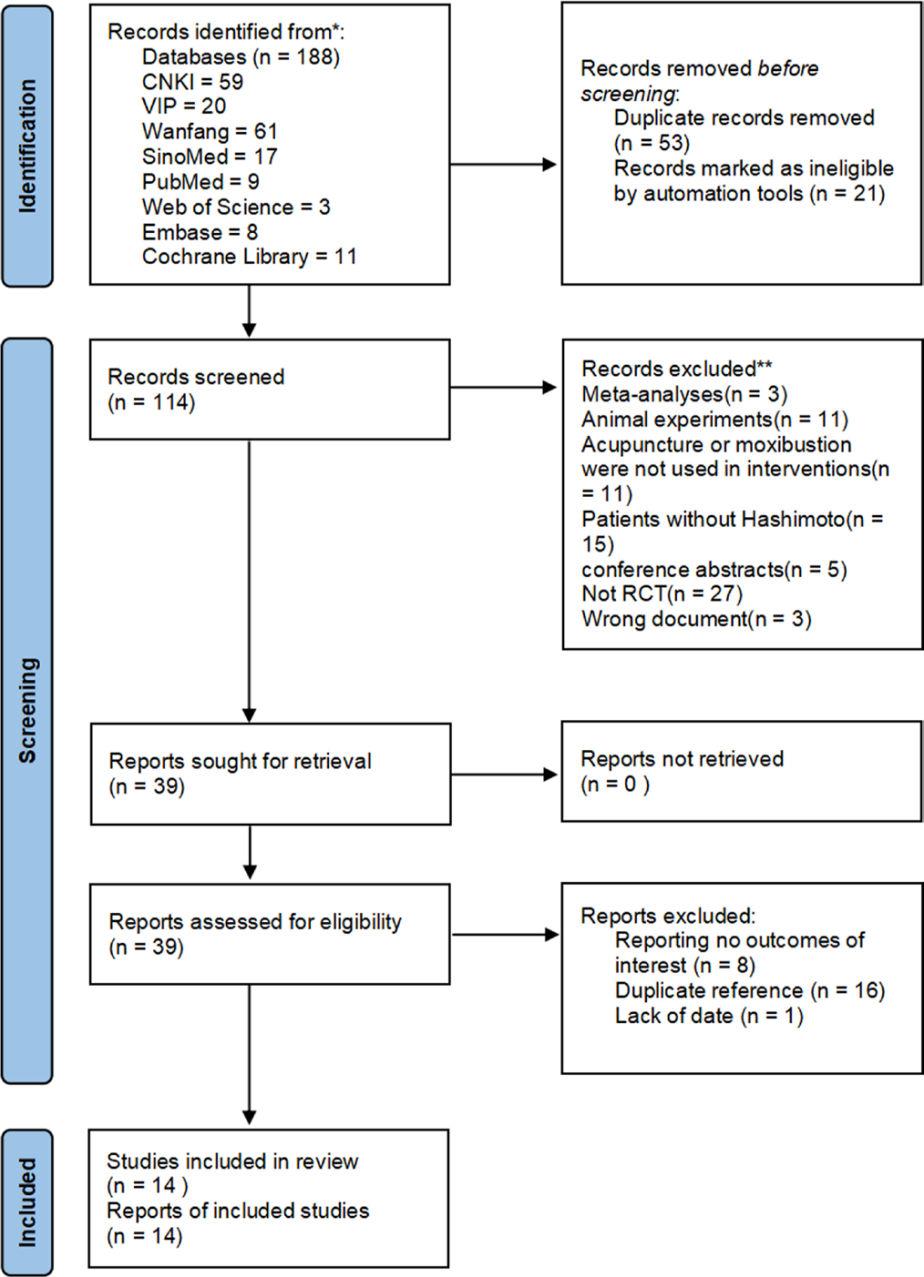
Flow chart of study selection.

The screening process of RCTs for meta-analysis (Fig. 1). After a preliminary systematic search, a total of 188 articles were retrieved, and 114 articles remained after the deletion of duplicate articles and records marked as ineligible by automation tools. Next, filter the title and abstract of the article. Excluded papers included 27 non-RCTs, 5 conference abstracts, 3 meta-analysis papers, 15 intervention object error and, 3 wrong document and 11 animal studies papers. Another 11 papers did not use acupuncture as an intervention group and were excluded. We screened the full text of the remaining 39 papers and excluded another 16 duplicate papers, 8 papers without the outcome indicators we needed, and 1 paper with incomplete data. Finally, 14 papers were included in this study.

### 3.1. Characteristics of included studies

Table 1 describes the detailed features of the literature included. The papers were published between 1987 and 2023. All of these studies were conducted in China. A total of 14 literatures were included in our study, among which 98 patients (the largest number of cases) were selected in the study of Ren Zhenxue et al^[14]^ while only 58 patients (the smallest number of cases) were selected in the study of Wang Xuze et al.^[15]^ Six of the papers studied Hashimoto thyroiditis with hypothyroidism,^[3,14,16–19]^ the other 8 literatures studied patients with Hashimoto thyroiditis.^[15,20–26]^ In the observation group, the treatment method was acupuncture plus levothyroxine sodium tablets,^[3,14,15,17–21,23,24]^ the other 4 are acupuncture.^[16,22,25,26]^ The point locations of 6 literatures were RN17, RN12, RN4, DU14, BL23 and DU4,^[16,20–23,26]^ in one of the literatures, ST36 was also taken on this basis,^[16]^ 2 of them took RN22.^[21,23]^ Point selection locations of the 3 literature were ST36, SP6, KI3, ST40, LI4, and LR3,^[3,14,18]^ one of them took the names RN17, RN12, RN4, RN22.^[18]^ Point locations of 1 study were RN4, BL23, ST36, KI1, BL20 and BL24.^[17]^ One is BL23, DU4, BL20, ST40, LR3.^[19]^ One is ST36, LI4, LI11, LI14, LI15 and ST9.^[15]^ Another study selected points ST36, SP6, KI3, LR3, GB34, SP9, LI11, SJ5 and LU7.^[25]^

**Table 1:** Characteristics of the included studies.

First author	Yr	Participants	Intervention methods		Number of patients (Intervention/control)	Treatment duration	Gender	Age	JADAD	Outcomes
			Observation group	Control group			(Male/Female)	(Intervention/control)		
Xia Yong	2012	HT	acupuncture + levothyroxine sodium tablet	levothyroxine sodium tablet	(40/40)	1 mo	10/75	47.15 ± 13.18/46.45 ± 13.85	5	FT3, FT4
Wang Xiaoyan	2003	HT&hypothyroidism	acupuncture	levothyroxine sodium tablet	(34/32)	6 mo	34/32	26~53/26~51	4	TSH, TGA, MCA, Iodine uptake rate
Cui Yunhua	2020	HT	acupuncture + levothyroxine sodium tablet	levothyroxine sodium tablet	(31/31)	3 mo	8/49	46 ± 13/47 ± 13	4	FT3, FT4, TSH, HADS-A, HADS-D
Hu Guosheng	1992	HT	acupuncture	levothyroxine sodium tablet	(35/32)	2 mo	4/63	19-66/19-66	3	TGA, MCA
Fang Zhenwei	2018	HT&hypothyroidism	acupuncture + levothyroxine sodium tablet	levothyroxine sodium tablet	(30/30)	1 mo	3/57	26~53/27~53	4	TGA, MCA
Hong Xiuyu	2020	HT&hypothyroidism	acupuncture + levothyroxine sodium tablet	levothyroxine sodium tablet	(37/38)	3 mo	22/53	43.11 ± 12.95/41.53 ± 12.56	4	TPOAb, TGAb, FT3, FT4, TSH
Wu Rui	2021	HT&hypothyroidism	acupuncture + levothyroxine sodium tablet	levothyroxine sodium tablet	(39/39)	3 mo	49/29	42.86 ± 5.63/42.33 ± 5.34	4	TPOAb, TGAb, FT3, FT4, TSH
Lin Shuting	2017	HT&hypothyroidism	acupuncture + levothyroxine sodium tablet	levothyroxine sodium tablet	(33/32)	3 mo	5/55	35.27 ± 9.39/34.87 ± 9.16	6	TPOAb, TGAb, FT4, TSH
Zheng Haidan	2019	HT	acupuncture + levothyroxine sodium tablet	levothyroxine sodium tablet	(45/54)	3 mo	13/89	44.61 ± 4.96/42.78 ± 4.04	6	TPOAb, TGAb, FT3, FT4, TSH
Wang Shanze	2022	HT	acupuncture + levothyroxine sodium tablet	levothyroxine sodium tablet	(29/29)	4 mo	6/52	48.55 ± 14.51/42.52 ± 12.50	6	TPOAb, TGAb, FT3, FT4, TSH, HADS-A, HADS-D
Zhou Zhenkun	2013	HT	acupuncture + levothyroxine sodium tablet	levothyroxine sodium tablet	(40/40)	1 mo	N/A	N/A	3	FT3, FT4, TSH
Chen Zian	2019	HT	acupuncture + massage	levothyroxine sodium tablet	(33/33)	1 mo	13/47	37.65 ± 7.84/38.46 ± 8.09	5	TPOAb, TGAb, FT3, FT4, TSH
Ren Zhenxue	2022	HT&hypothyroidism	acupuncture + levothyroxine sodium tablet	levothyroxine sodium tablet	(49/49)	3 mo	60/38	43.27 ± 2.17/N/A	4	TPOAb, TGAb, FT3, FT4, TSH
Hu Guosheng	1987	HT	acupuncture	levothyroxine sodium tablet	(34/32)	1 mo	2/66	45 ± 9.09/N/A	3	TSH, TGA, MCA, Iodine uptake rate

FT3 = serum free triiodothyronine, FT4 = serum free thyroxine, HADS-A = hospital anxiety scale, HADS-D = hospital depression scale, HT = Hashimoto thyroiditis, MCA = serum anti-thyroid microsomal antibody, TGA = serum anti-thyroid peroxidase antibody, TGAb = thyroglobulin antibody, TPOAb = thyroid peroxidase antibody, TSH = thyroid stimulating hormone.

### 3.2. Primary outcomes

#### 3.2.1. TPOAb level.

**Figure 2.**
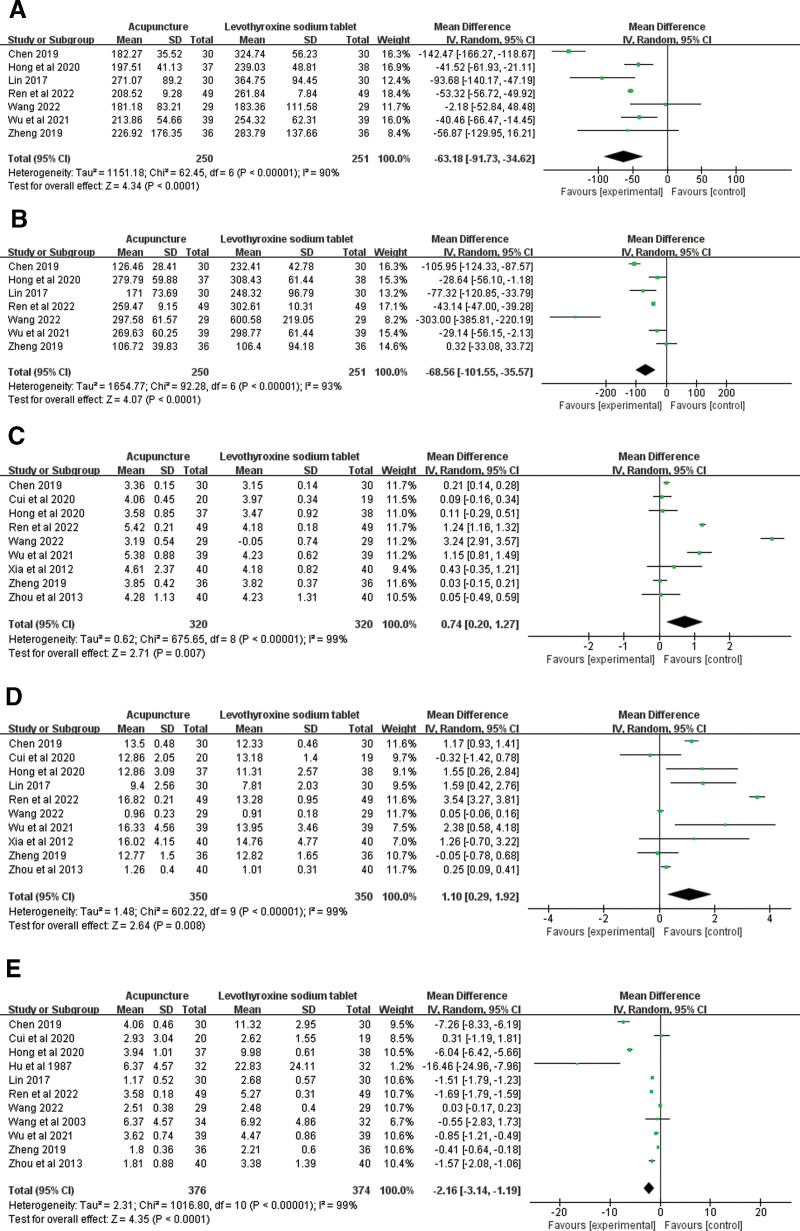
Forest plots of the effects of acupuncture on TPOAb (A) and TGAb (B), FT3 (C), FT4 (D), TSH (E). FT3 = serum free triiodothyronine, FT4 = serum free thyroxine, TGAb = thyroglobulin antibody, TPOAb = thyroid peroxidase antibody, TSH = thyroid stimulating hormone.

TPOAb content was reported in 7 literatures^[3,14,15,18,19,23,25]^ (Fig. 2A). Heterogeneity analysis showed a high degree of heterogeneity among the studies (*I^2^* = 90%, *P* < .0001), so meta-analysis was performed using random effects models. Meta-analysis showed that the improvement of TPOAb level in acupuncture group was significantly better than that in levothyroxine sodium tablets group (MD = −63.18, 95%CI = −91.73 to −34.62, *P* < .00001). Subgroup analysis was performed according to JADAD score, duration of treatment, and disease type. Heterogeneity was significantly reduced in the group treated for more than 1 month, suggesting that the duration of treatment may be the source of heterogeneity. The subgroup results are shown in Table 2. Heterogeneity was significantly reduced after 1 article^[25]^ was removed from the sensitivity analysis (*I^2^* = 44%, *P* = .11). The reason for the analysis may be different intervention methods. Most of the literature used acupuncture combined with levothyroxine sodium tablets for treatment, while acupuncture combined with massage was used in this literature. The funnel plot for this outcome indicator is A in Supplementary Figure 1, http://links.lww.com/MD/L799.

**Table 2:** Subgroup analysis of TPOAb and TGAb.

Subgroups	TPOAb							TGAb						
	Studies, n	Participants, n	*I* ^2^	Q-test	Mean difference	95% CI	*P*	Studies, n	Participants, n	*I* ^2^	Q-test	Mean difference	95% CI	*P*
JADAD grade														
1-4	3	251	6	0.35	−52.14	−57.22, −47.06	<.00001	3	251	1	0.36	−42.44	−46.71, −38.17	<.00001
5-7	4	250	89	<0.00001	−76.60	−143.70, −9.51	<.00001	4	250	95	<0.00001	−112.49	−191.74, −33.24	.005
Treatment duration														
<1 mo	2	120	70	0.07	−122.33	−169.41, −75.25	<.00001	2	120	29	0.24	−98.71	−123.10, −74.33	<.00001
>1 mo	5	381	33	0.20	−46.51	−58.16, −34.87	<.00001	5	381	91	<0.00001	−57.37	−95.32, −19.43	.003
Disease type														
Hashimoto thyroiditis	3	190	92	<0.00001	−69.35	−167.71,29.00	.17	3	190	96	<0.00001	−127.81	−237.95, −17.66	.02
Hashimoto thyroiditis with hypothyroidism	4	311	41	0.17	−51.14	−63.23,−39.06	<.00001	4	311	33	0.22	−41.13	−53.46, −28.80	<.00001

CI = confidence interval, TGAb = thyroglobulin antibody, TPOAb = thyroid peroxidase antibody.

#### 3.2.2. TGAb level.

TGAb content was reported in 7 literatures^[3,14,15,18,19,23,25]^ (Fig. 2B). Random effects models were used for meta-analysis due to the high heterogeneity among studies (*I*^2^ = 93%, *P* < .0001). Meta-analysis showed that the improvement of TGAb level in acupuncture group was significantly better than that in levothyroxine sodium tablets group (MD = −68.56, 95%CI = −101.55 to −35.57, *P* < .00001). Despite subgroup analysis based on JADAD score, duration of treatment, and type of disease, heterogeneity remained. Heterogeneity was significantly reduced in the treatment duration less than 1-month group and in the Hashimoto group with hypothyroidism, suggesting that the duration of treatment and type of disease may be the source of heterogeneity. The subgroup results are shown in Table 2. A sensitivity analysis showed that heterogeneity remained constant. The funnel plot for this outcome indicator is B in Supplementary Figure 1, http://links.lww.com/MD/L799.

#### 3.2.3. FT3 level.

FT3 content was reported in 9 literatures^[3,14,15,18,20,21,23–25]^ (Fig. 2C). Random effects models were used for meta-analysis due to the high heterogeneity among studies (*I*^2^ = 99%, *P* = .007). Meta-analysis showed that FT3 levels in the acupuncture group were significantly better than those in the levothyroxine sodium tablets group (MD = 0.74, 95%CI = 0.20 to 1.27, *P* < .00001). Heterogeneity persisted after doing subgroup analyses of acupuncture method, JADAD score, treatment duration and disease type, and dose. Subgroup analyses could not explain the heterogeneity. The subgroup results are shown in Table 3. A sensitivity analysis showed that heterogeneity remained constant. The funnel plot for this outcome indicator is C in Supplementary Figure 1, http://links.lww.com/MD/L799.

**Table 3:** Subgroup analysis of FT3, FT4.

Subgroups	FT3							FT4						
	Studies, n	Participants, n	*I* ^2^	Q-test	Mean difference	95% CI	*P*	Studies, n	Participants, n	*I* ^2^	Q-test	Mean difference	95% CI	*P*
Therapies														
Acupuncture	4	328	98	<0.00001	0.63	−0.12,1.38	.10	4	328	99	<0.00001	1.51	−0.67,3.68	.18
Cake-separated moxibustion	2	177	100	<0.00001	1.66	−1.42,4.75	.29	2	97	0	0.51	0.05	−0.06,0.15	.39
JADAD grade														
1-4	5	370	96	<0.00001	0.54	−0.07,1.16	.08	5	370	99	<0.00001	1.47	−0.51,3.45	.15
5-7	4	190	99	<0.00001	1.15	−0.11,2.40	.07	4	250	95	<0.00001	0.63	−0.17,1.43	.12
Treatment duration														
<1 mo	3	218	93	<0.00001	0.48	−0.18,1.13	.15	4	278	94	<0.00001	1.08	0.31,1.85	.006
>1 mo	5	342	99	<0.00001	0.94	0.06,1.82	.04	5	342	99	<0.00001	0.96	−1.01,2.94	.34
Disease type														
Hashimoto thyroiditis	5	309	99	<0.00001	0.72	−0.09,1.53	.08	5	309	94	<0.00001	0.31	−0.14,0.77	.18
Hashimoto thyroiditis with hypothyroidism	3	251	93	<0.00001	0.86	0.26,1.45	<.00001	4	311	84	0.0002	2.34	1.05,3.64	.0004

CI = confidence interval, FT3 = serum free triiodothyronine, FT4 = serum free thyroxine.

#### 3.2.4. FT4 level.

FT4 content was reported in 10 literatures^[3,12,13,16–19,21–23]^ (Fig. 2D). Heterogeneity analysis showed a high degree of heterogeneity among the studies (*I^2^* = 99%, *P* = .008), so meta-analysis was performed using random effects models. Meta-analysis showed that FT4 level in the acupuncture group was significantly better than that in the levothyroxine sodium tablets group (MD = 1.10, 95%CI = 0.29 to 1.92, *P *< .00001). Subgroup analysis was performed according to acupuncture method, JADAD score, treatment duration and disease type, heterogeneity remained. The subgroup results are shown in Table 3. A sensitivity analysis showed that heterogeneity remained constant. The funnel plot for this outcome indicator is C in Supplementary Figure 1, http://links.lww.com/MD/L799. The funnel plot for this outcome indicator is D in Supplementary Figure 1, http://links.lww.com/MD/L799.

#### 3.2.5. TSH level.

TSH content was reported in 10 literatures^[3,14–16,18,19,21,23–25]^ (Fig. 2E). Heterogeneity analysis showed a high degree of heterogeneity among the studies (*I^2^ *= 99%, *P* < .0001), so meta-analysis was performed using random effects models. Meta-analysis showed that the improvement of TSH level in acupuncture group was significantly better than that in levothyroxine sodium tablets group (MD = −2.16, 95%CI = −3.14 to −1.19, *P* < .00001). Subgroup analysis was performed according to acupuncture method, JADAD score, treatment time, disease type, and publication date, heterogeneity remained. Group heterogeneity was significantly reduced after 2010, suggesting that publication date may be the source of heterogeneity. The subgroup results are shown in Table 4. A sensitivity analysis showed that heterogeneity remained constant. The funnel plot for this outcome indicator is E in Supplementary Figure 1, http://links.lww.com/MD/L799.

**Table 4:** Subgroup analysis of TSH.

Subgroups	TSH						
	Studies, n	Participants, n	*I* ^2^	Q-test	Mean difference	95% CI	*P*
Therapies							
Acupuncture	4	328	97	<0.00001	−1.13	−1.88, −0.37	.003
Cake-separated moxibustion	3	169	86	0.0007	−2.87	−7.29,1.55	.20
JADAD grade							
1-4	7	494	99	<0.00001	−1.56	−2.90, −0.23	.02
5-7	4	256	98	<0.00001	−3.50	−5.38, −1.62	.0003
Treatment duration							
<1 mo	5	336	98	<0.00001	−0.76	−0.90, −0.61	<.00001
>1 mo	6	414	99	<0.00001	−1.73	−1.82, −1.64	<.00001
Disease type							
Hashimoto thyroiditis	6	373	98	<0.00001	−0.40	−0.54, −0.26	<.00001
Hashimoto thyroiditis with hypothyroidism	5	377	99	<0.00001	−1.86	−1.94, −1.77	<.00001
Date of publication							
<2010	9	626	99	<0.00001	−2.58	−3.68, −1.49	<.00001
≥2010	2	124	0	0.62	0.03	−0.17,0.23	.80

CI = confidence interval, TSH = thyroid stimulating hormone.

### 3.3. The secondary outcome

#### 3.3.1. MCA level.

**Figure 3.**
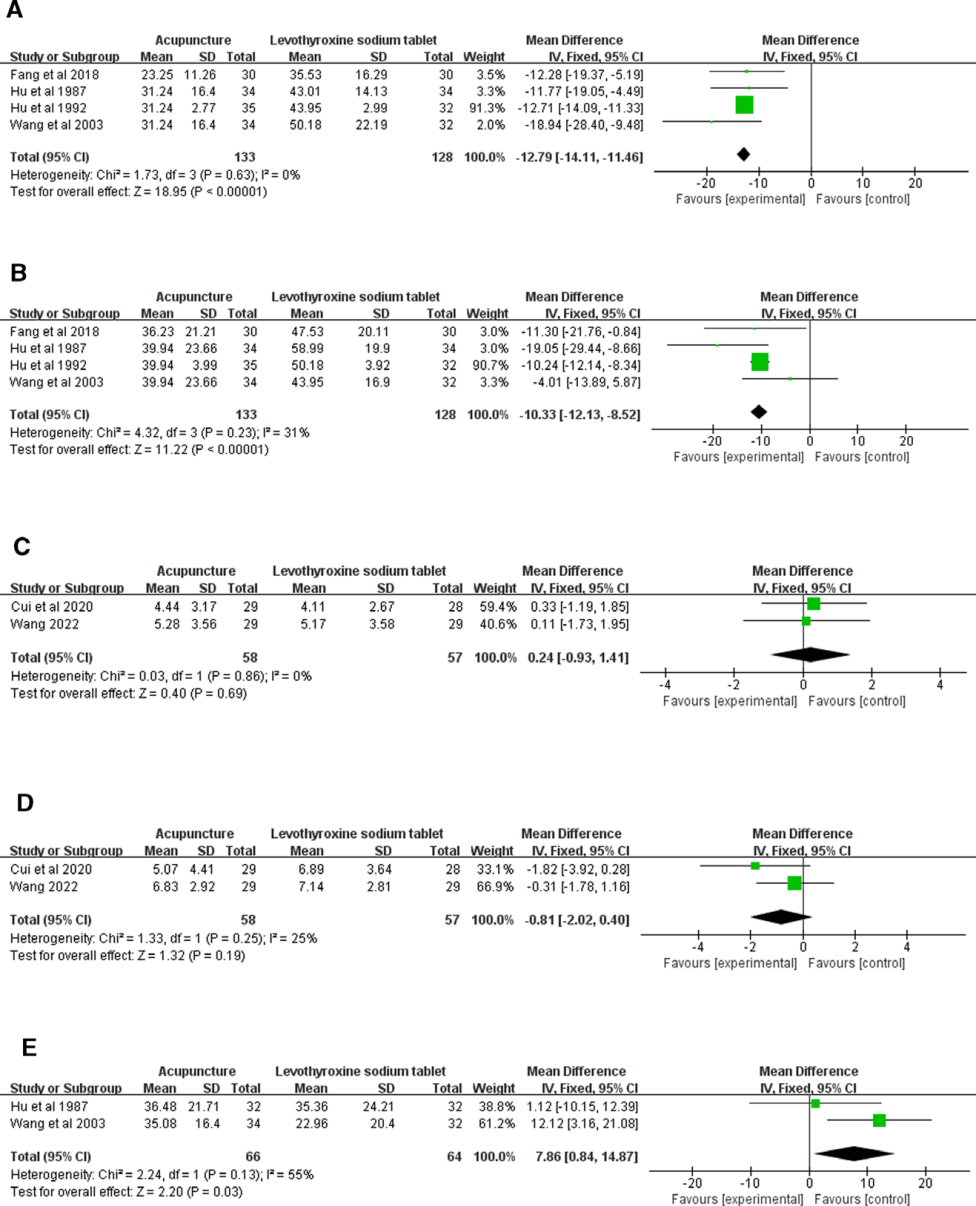
Forest plots of the effects of acupuncture on MCA (A)and TGA (B), HADS-A (C), HADS-D (D) and Iodine uptake rate (E). HADS-A = hospital anxiety scale, HADS-D = hospital depression scale, TGA = serum anti-thyroid peroxidase antibody.

MCA content was reported in 4 literatures^[16,17,22,26]^ (Fig. 3A). Meta-analysis showed that there was no significant difference between acupuncture and levothyroxine sodium tablets in reducing MCA content (MD = −12.79, 95%CI = −14.11 to −11.46, *P *= .63), showed low heterogeneity (*I*^2^ = 0%, *P* < .00001). Subgroup analysis was performed according to treatment duration. The subgroup results are shown in Table 5. The funnel plot for this outcome indicator is A in Supplementary Figure 2, http://links.lww.com/MD/L800.

**Table 5:** Subgroup analysis of MCA, TGA.

Subgroups	MCA							TGA						
	Studies, n	Participants, n	*I* ^2^	Q-test	Mean difference	95% CI	*P*	Studies, n	Participants, n	*I* ^2^	Q-test	Mean difference	95% CI	*P*
Treatment duration														
<1 mo	2	128	0	0.92	−12.03	−17.11, −6.96	<.00001	2	128	6	0.30	−15.20	−22.57, −7.83	<.00001
>1 mo	2	133	39	0.20	−12.84	−14.21, −11.47	<.00001	2	133	32	0.22	−10.02	−11.88, −8.16	<.00001

CI = confidence interval, MCA = serum anti-thyroid microsomal antibody, TGA = serum anti-thyroid peroxidase antibody.

#### 3.3.2. TGA level.

The content of TGA was reported in 4 literatures^[16,17,22,26]^ (Fig. 3B). Meta-analysis showed no significant difference between acupuncture and levothyroxine sodium tablets in reducing TGA content (MD = −10.33, 95%CI = −12.13 to −8.52, *P* = .23). Showed low heterogeneity (*I*^2^ = 31%, *P* < .00001). Subgroup analysis was performed according to treatment duration. The subgroup results are shown in Table 5. The funnel plot for this outcome indicator is B in Supplementary Figure 2, http://links.lww.com/MD/L800.

#### 3.3.3. HADS-A.

HADS-A scores have been reported in 2 literatures^[15,21]^ (Fig. 3C). Meta-analysis showed that there was no significant difference between acupuncture and levothyroxine sodium tablets in reducing the HADS-A score (MD = 0.24, 95%CI = −0.93 to 1.41, *P *= .86), showed low heterogeneity (*I*^2^ = 0%, *P* = .69). The funnel plot for this outcome indicator is C in Supplementary Figure 2, http://links.lww.com/MD/L800.

#### 3.3.4. HADS-D.

HADS-D scores were reported in 2 literatures^[15,21]^ (Fig. 3D). Meta-analysis showed no significant difference between acupuncture and levothyroxine sodium tablets in reducing HADS-D score (MD = −0.81, 95%CI = −2.02 to 0.40, *P *= .25), showed low heterogeneity (*I*^2^ = 25%, *P* = .19). The funnel plot for this outcome indicator is E in Supplementary Figure 2, http://links.lww.com/MD/L800.

#### 3.3.5. Iodine uptake rate.

The iodine uptake rate was reported in 2 literatures^[16,26]^ (Fig. 3E). Meta-analysis showed that there was no significant difference between acupuncture and levothyroxine sodium tablets in improving iodine uptake rate (MD = 7.86, 95%CI = 0.84 to 14.87, *P* = .13), showed low heterogeneity (*I*^2^ = 55%, *P* = .03). The funnel plot for this outcome indicator is E in Supplementary Figure 2, http://links.lww.com/MD/L800.

### 3.4. Assessment of risk of bias

**Figure 4.**
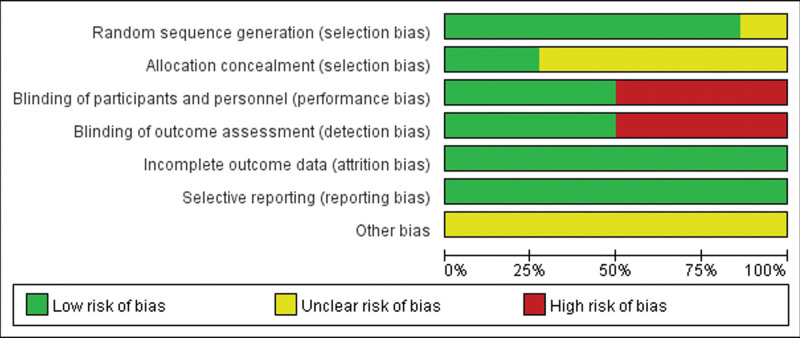
RCT risk of bias summary for included Randomized Controlled Trial. RCTs = randomized controlled trials.

The purpose of funnel plots is to show whether the included studies have significant publication bias (Fig. 4). Five articles^[15,19,20,23,25]^ do not describe the specific method of double blindness. Nine papers^[3,14,16–18,21,22,24,26]^ did not use double blindness, so both types are rated as high risk. Nine articles^[3,14,16–18,20–22,24–26]^ do not describe the method of distributing concealment, but the rest of the articles are distributed by the characteristics of hidden opaque envelopes or hidden bottles. Three papers^[22,24,26]^ did not describe the randomization method, which was assessed as unclear. In the rest of the study, 11 articles^[3,14–21,23,25]^ were grouped according to a random number table.

## 4. Discussion

HT is considered to be the most common autoimmune disease.^[27]^ It is considered a typical, organ-specific, autoimmune disease, characterized by autoimmune-mediated destruction of the thyroid gland.^[28]^ Patients with Hashimoto thyroiditis and overt hypothyroidism are generally treated with lifelong thyroid hormone therapy.^[29]^ The drug can significantly improve the patient thyroid function, but long-term use may induce cardiovascular system, bone metabolism and other related complications. Acupuncture treatment has few side effects and can improve thyroid function and antibody levels in HT patients to a certain extent. Its mechanism of action is mainly related to the adjustment of hypothalamic-pituitary-thyroid axis function and immune capacity of the body.

The disease and traditional Chinese medicine “gall disease” is very similar, in the late course of the spleen and kidney deficiency. Traditional Chinese medicine combined with the treatment ideas of deficiency and fullness, Zang-Fu organs and traditional Western medicine, effectively alleviated clinical symptoms, highlighting the advantages of treatment. In this study, a total of 14 randomized controlled trials were included for meta-analysis to determine whether acupuncture was superior to levothyroxine sodium tablets alone in terms of therapeutic efficacy, especially in regulating hormone content. Aconite cake-separated moxibustion at Guanyuan (CV4) and Mingmen (GV4) combined with oral administration of Euthyrox can improve clinical symptoms and thyroid function in patients of Hashimoto thyroiditis, which is better than simple oral administration of Euthyrox.^[6]^

Ten independent outcome markers were used for comparison. The main outcome markers included TPOAb, TGAb, FT3, FT4 and TSH. Secondary outcome measures included MCA, TGA, HADS-A, HADS-D and Thyroid iodine uptake. The results showed that acupuncture and moxibustion could regulate the levels of FT3, FT4, TSH, TPOAb and TGAb. This is consistent with the meta-analysis results of Wu Rui et al^[3]^ Yuan Qing^[30]^ acupuncture and moxibustion can reduce the volume of the enlarged thyroid gland, applied the “three needles of protrusion” to treat the nodular goiter, namely, the celestial protrusion, the support protrusion, and the water protrusion, and achieved good results in the treatment of nodular goiter. Acupuncture of these acupoints can regulate the local blood supply of the thyroid, increase the blood flow of the artery, inhibit the vagus nerve, reduce the secretion of TSH, and promote the reduction of the enlarged thyroid gland.^[9]^ The therapeutic effect of acupuncture combined with levothyroxine sodium tablets is superior to that of levothyroxine sodium tablets alone, and it can treat both symptoms and root causes according to the etiology and solve the problem that single hormone supplement therapy is difficult to improve the thyroid antibody concentration. However, there was no significant difference between acupuncture and levothyroxine sodium tablets in improving MCA, TGA, HADS scores and iodine uptake rate.

Our meta-analysis showed that acupuncture and moxibustion had a significant regulating effect on the contents of TPOAb, TGAb, TSH, FT3 and FT4. Acupuncture and moxibustion at ST36, LI4, SP6, BL20 and CV4 points could make patients progress to normal TSH levels, which was consistent with the results of Nair PMK.^[31]^ Zhang Yuying^[32]^ study found that moxibustion on Mingmen and Guanyuan points can reduce the contents of TPOAb and TGAb, combined with Shanzhong to regulate Qi, Zhongwan Temperature to replenish Qi, and Dazhui and Shenshu points to strengthen the strength of Yang Qi. Xia Yong^[6]^ study found that the serum free thyroxine index (FT4) content increased significantly after moxibustion treatment, indicating that moxibustion can regulate the content of FT4.

There are still many shortcomings in this study. The bias risk of some RCTS included is high and the literature quality is low, so the results of this systematic evaluation are still uncertain. Future randomized controlled trials of moxibustion in the treatment of HT should specify the method of random sequence generation, as well as the method of assigning concealment.

## 5. Conclusion

Compared with levothyroxine sodium tablets, acupuncture can significantly improve the levels of TPOAb, TGAb, FT3, FT4 and TSH in HT patients, but there is no significant difference between acupuncture and levothyroxine sodium tablets in improving the levels of MCA, TGA, HADS score and iodine uptake rate. In future studies, large samples and high-quality randomized controlled trials should be conducted.

## Author contributions

**Conceptualization:** Xiaohui Wang, Xinying Zhu.

**Data curation:** Xiaohui Wang, Yu Li, Zhicheng Dai, Tongxia Zhan.

**Formal analysis:** Yu Li, Zhicheng Dai.

**Investigation:** Limin Ma, Xinying Zhu.

**Methodology:** Xiaohui Wang, Limin Ma, Xinying Zhu.

**Resources:** Xinying Zhu.

**Supervision:** Hai Xie.

**Validation:** Hai Xie, Tongxia Zhan.

**Visualization:** Tongxia Zhan.

**Writing – original draft:** Xiaohui Wang.

## Supplementary Material




