# Supplementary figures and images for: Effect of acupuncture on Hashimoto thyroiditis: A systematic review and meta-analysis
Source: Medicine (Baltimore). 2024 Mar 1;103(9):e37326. doi: 10.1097/MD.0000000000037326 (PMC10906624; doi:10.1097/MD.0000000000037326)

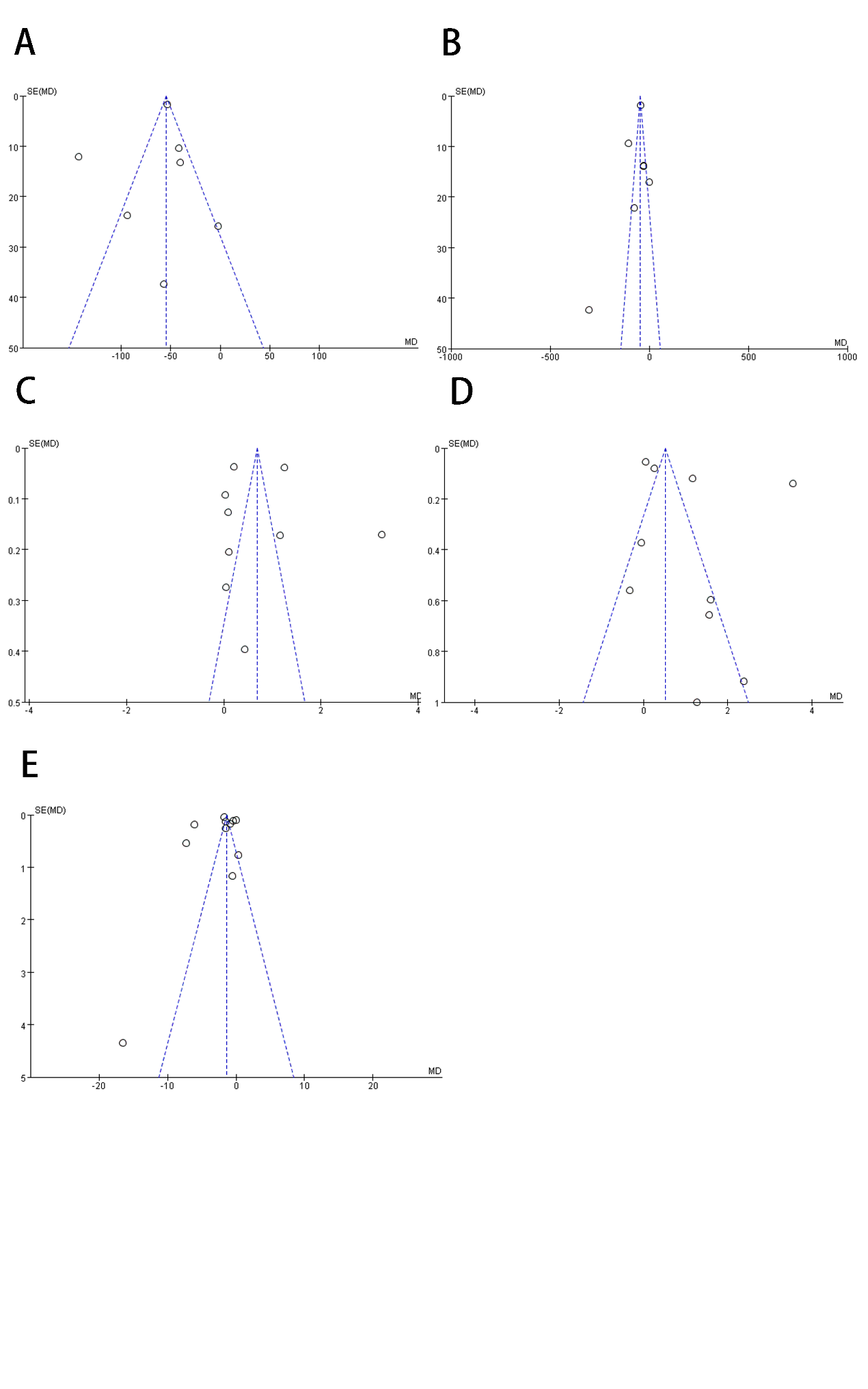

Supplement: Supplementary file 1 [file medi-103-e37326-s001.doc]

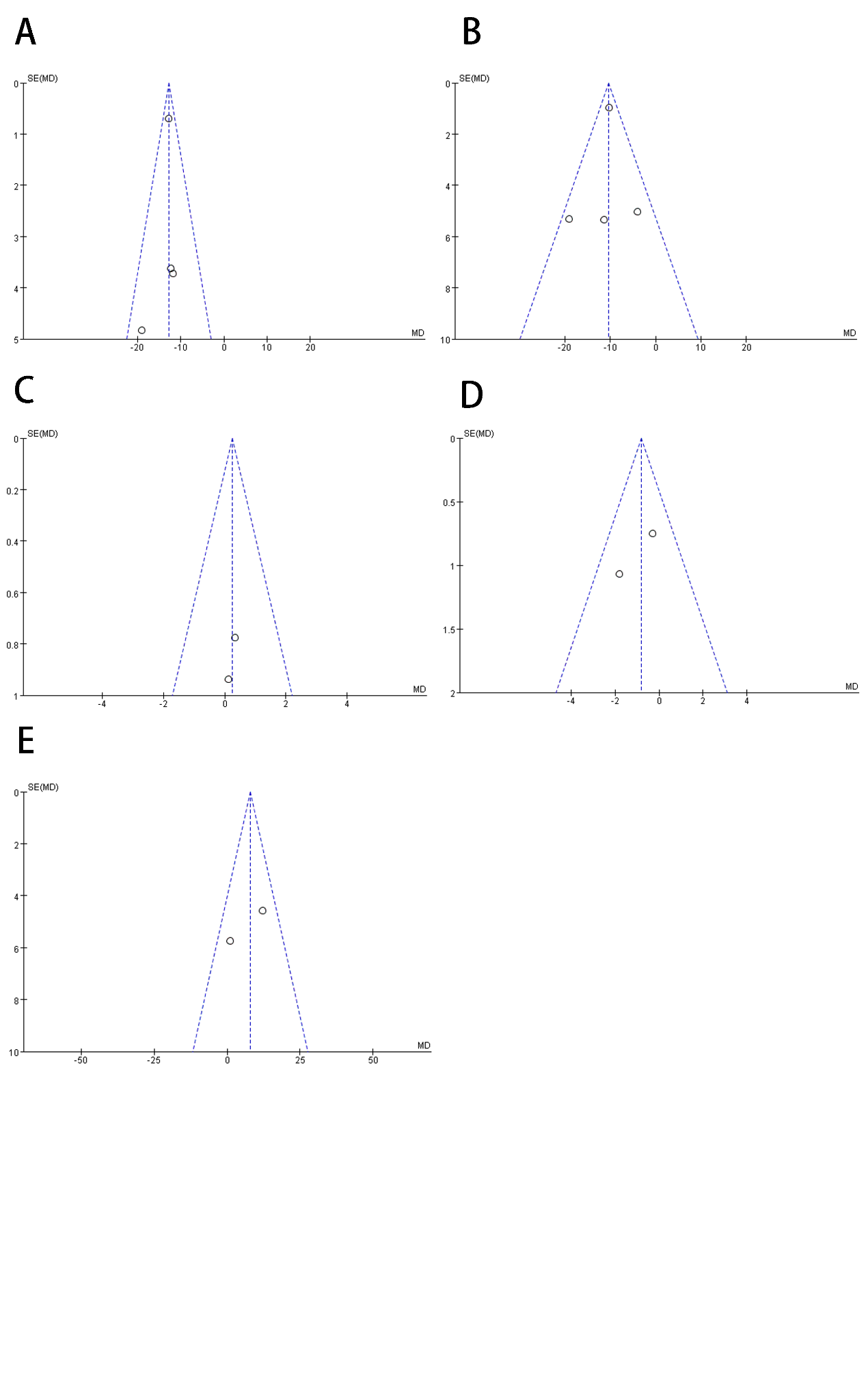

Supplement: Supplementary file 2 [file medi-103-e37326-s002.doc]
